# Development of a Qualitative Data Analysis Codebook for Arterial Hypertension and Type-2-Diabetes Integrated Care Evaluation

**DOI:** 10.5334/ijic.7691

**Published:** 2024-03-22

**Authors:** Črt Zavrnik, Nataša Stojnić, Majda Mori Lukančič, Monika Martens, Katrien Danhieux, Savina Chham, Matic Mihevc, Tina Virtič Potočnik, Zalika Klemenc Ketiš, Josefien van Olmen, Antonija Poplas Susič

**Affiliations:** 1 Primary Healthcare Research and Development Institute, Community Health Centre Ljubljana, Metelkova ulica 9, SI-1000 Ljubljana, Slovenia; 2 Department of Family Medicine, Faculty of Medicine, University of Ljubljana, Poljanski nasip 58, SI-1000 Ljubljana, Slovenia; 3 Institute of Tropical Medicine Antwerp, Nationalestraat 155, BE-2000 Antwerp, Belgium; 4 University of Antwerp, Department of Family Medicine and Population Health, Prinsstraat 6, BE-2000 Antwerp, Belgium; 5 National Institute of Public Health, Cambodia, Boeung Kok 2 quarter, KH-120408 Phnom Penh, Cambodia; 6 Primary Healthcare Centre Trebnje, Goliev trg 3, SI-8210 Trebnje, Slovenia; 7 Primary Healthcare Centre Slovenj Gradec, Partizanska pot 16, SI-2380 Slovenj Gradec, Slovenia; 8 Department of Family Medicine, Faculty of Medicine, University of Maribor, Taborska ulica 8, SI-2000 Maribor, Slovenia

**Keywords:** arterial hypertension, type-2-diabetes, codebook, qualitative analysis

## Abstract

**Introduction:**

Non-communicable diseases, such as arterial hypertension (HTN) and type-2 diabetes (T2D), pose a global public health problem. Integrated care with focus on person-centred principles aims to enhance healthcare quality and access. Previous qualitative research has identified facilitators and barriers for scaling-up integrated care, however the lack of standardized terms and measures hinder cross-country comparisons. This paper addresses these gaps by presenting a generic codebook for qualitative research on integrated care implementation for HTN and T2D.

**Description:**

The codebook serves as a tool for deductive or deductive-inductive qualitative analysis, organizing concepts and themes from qualitative data. It consists of nine first level and 39 second level themes. First level codes cover core issues; and second level codes provide detailed insights into facilitators and barriers.

**Discussion:**

This codebook is more widely applicable than previously developed tools because it includes a broader scope of stakeholders across micro, meso, and macro levels, and the themes being derived from highly diverse health systems across high- and low-income countries.

**Conclusion:**

The codebook is a useful tool for implementation research on integrated care for HTN and T2D at global scale. It facilitates cross-country learning, contributing to improved implementation, scale-up and outcomes.

## Introduction, Comprising Background and Problem Statement

Non-communicable diseases, including arterial hypertension (HTN) and type-2-diabetes (T2D), increasingly represent a major public health problem worldwide as a result of rapid urbanization, aging populations, and the global spread of unhealthy lifestyles [1]. This phenomenon is observed in low- and middle-income countries (LMICs) and high-income countries [2,3]. The acknowledgment of integrated care as a viable approach to address the growing challenge of non-communicable diseases involves structured initiatives to deliver coordinated, proactive, person-centred, and multidisciplinary care [4]. Although integrated care models have been implemented in various health systems worldwide [5], their widespread implementation has been hindered by barriers that depend mainly on the macro context of each country (e.g., cultural resistance, type of health system, laws and regulations) [6,7,8].

Implementation research plays a crucial role in the scale-up of integrated care for non-communicable diseases. The implementation of integrated care and the identification of facilitators and barriers in different settings have been extensively studied in previous research using quantitative, qualitative, and mixed-methods [9,10,11,12,13,14,15,16]. The wide variety of terms, measures, and infrequent use of common sets in literature related to integrated care of chronic conditions makes it difficult to compare different implementation research projects [17]. For instance, distinct codes may occasionally encompass identical concepts, (e.g., *Identification* and *Disease diagnosis* for identification and determination of a specific medical condition or illness). Conversely, identical codes may signify divergent concepts, exemplified by *Education*, which may pertain to the training of healthcare professionals for the management of patients with a particular disease or, alternately, to the education of patients about a specific disease and its treatment. Additionally, concepts may be embedded within various themes across different papers, such as the incorporation of *Access to medication* into themes like *Barriers to diabetes management* or *Pharmaceutical*. [12,13,14,16].

To overcome above barriers, large multi-country projects such as *Innovating care for people with multiple chronic conditions in Europe* (ICARE4EU) [18] and *Sustainable integrated care models for multi-morbidity delivery, financing and performance* (SELFIE) [19] have contributed to common language and frameworks, such as SELFIE-framework [20]. However, there are two major limitations. First, the (slow) evolution of frameworks into tools for collaborative work and comparison, such as core outcome sets, quality checklists for reporting, and repositories of research tools and data. While repositories for epidemiologic and clinical data are becoming more common, they are still exceptional for qualitative data. Our exploration of existing repositories [21,22] for integrated care of HTN and T2D revealed only a handful of codebooks [23,24,25,26]. Second, existing tools and instruments have largely been applied in European and North American settings. If we seek global progress, we need to co-develop tools with researchers from both high-income countries and LMICs to address implementation needs in their contexts.

This paper addresses both gaps by presenting a tool – a generic codebook for qualitative research on the implementation of integrated care for two major non-communicable diseases (HTN and T2D) developed by research teams in one LMIC and two high-income countries.

## Methods

The research methodology presented has been developed in the context of the SCUBY (SCale-Up diaBetes and hYpertension care) project – an international project that assesses the current status of implementation and then identifies pathways to scale-up the integrated care package for patients with HTN and T2D at the primary health care level. SCUBY is implemented in three countries with different contexts – Cambodia, a LMIC with an evolving health system; Slovenia, a high-income country with a centralized health system and in Belgium, a high-income country with a fragmented health system [27]. The method is built upon a deductive-inductive approach [28,29], in which both field-collected data in the three countries and themes from literature contributed to the codebook, in either consecutive or parallel phases.

### Objective

This paper aims to create a comprehensive codebook for qualitative research on the implementation of integrated care for HTN and T2D. A codebook is a structured document detailing the set of codes employed to categorize and label diverse segments of qualitative data in the coding process. Functioning as a guide, this document ensures researchers consistently apply codes to the data. A code is often a word or short phrase that symbolically assigns a summary, salient, essence-capturing, or attribute to a piece of data. A theme denotes a recurring idea or pattern that arises from the coded data, representing higher-order constructs that capture the essence by grouping related codes together [29].

### Data

Data came from participants representing all three levels of the WHO multilevel qualitative framework [30]:

- Micro level (patients and health care workers): inclusion criteria for patients with HTN and T2D were an age of 65 years or more (Slovenia and Belgium) or age of 40 years or more (Cambodia) and the ability to communicate verbally; for health workers, inclusion criteria were representation of different geographic locations (urban and rural) and coverage of all primary health care team members, depending on the health system studied (e.g., general practitioners, nurse practitioners, practice nurses, and community health workers);
- Meso (community and health care organisations) and macro (regulatory, financial, professional, and scientific stakeholders) levels: participants were purposively selected based on their relevance for integrated care and their role in the health system.

### Development of the Codebook

**Figure 1:**
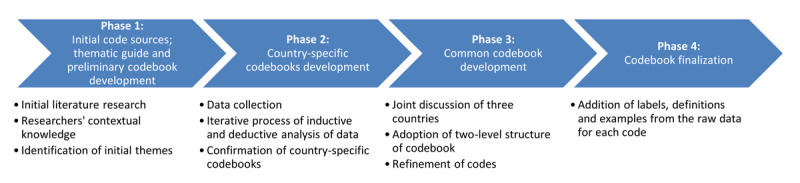
The four-phase process of the codebook for the evaluation of integrated care of arterial hypertension and type-2-diabetes.

The codebook was developed by the researchers of the three implementation countries (ČZ, NS, MML, MM, KD, SC) and two researchers with a strong methodological background (APS, JVO) in four phases (Figure 1).

#### Phase 1: initial code sources; thematic guide and preliminary codebook development

In the first phase of the codebook development, which lasted from January until April 2019, researchers from all three countries worked together under the guidance of the experienced researchers (APS, JVO) to develop a common thematic guide for the interviews and focus groups. The preliminary themes were based on initial literature review and contextual knowledge and led to a preliminary codebook. Across the country teams, an up-to four-level structure of the preliminary codebook was agreed upon. The detailed thematic guides are presented in Appendices 1 and 2.

#### Phase 2: country-specific codebooks development

In the second phase, which lasted from April 2019 until September 2020, each of the three countries conducted interviews and focus groups separately. The preliminary codebook (phase 1) was thus field-tested in data collection on the evaluation of implementation of integrated care for HTN and T2D in semi-structured interviews and focus groups in all three countries. A total of 84 in-depth, semi-structured interviews and 29 focus groups were conducted. All interviews and focus groups were conducted face-to-face by at least two researchers (ČZ, NS, MML, KD, SC). They lasted 30-90 minutes and were audio recorded. Data were collected until data saturation on the research topic was achieved. The characteristics of the data collection are presented in Table 1 and the detailed selection of participants is described in Appendix 3.

**Table 1:** The characteristics of the data collection. HTN – arterial hypertension; T2D – type-2-diabetes.


PARTICIPANTS LEVEL	CAMBODIA	SLOVENIA	BELGIUM

Micro	14 focus groups: four with patients with HTN and/or T2D; five with health care workers; five with community health workers	15 focus groups: seven with patients with HTN and T2D; eight with health care workers	not performed

Meso	15 interviews	11 interviews	15 interviews

Macro	18 interviews	12 interviews	13 interviews

Time period of data collection:	June 2019–September 2019	May 2019–April 2020	April 2019–September 2020


In each country, at least two independent researchers (ČZ, NS, MML, SC, KD, MM) analysed each transcript, using both an inductive (bottom-up; starting from the data) and deductive (top-down; based on initial codebook) approach. The concept of open-coding (identifying themes, comparing, and categorising concepts or statements) was used in the analysis [29]. In this iterative process, which was led by a lead researcher in each research group (APS, JVO, Por Ir), the designed codebooks were refined (some codes were changed, removed, or added). After several iterations, with no new themes emerging, the codebooks were adopted as valid representations of the data. The final country-specific codebooks were presented in English to allow comparison and had up to a four-level structure. The first level included identified core themes that generally allow for coverage of all issues that arise during the coding process. The second- to fourth-level themes allow further systematization of recognized themes in a tree structure with an aim of in-depth insight into facilitators and barriers in a specific health care system.

#### Phase 3: common codebook development

After completion of the country-specific codebooks, team members from all three countries met for joint online discussions to compare the phase 2 country specific codebooks in order to come to an adapted version of a common codebook. Several online sessions were organised and several e-mail correspondences were shared in this phase, which lasted since November 2019 until March 2020. These sessions were led by researchers experienced in qualitative methodology (APS, JVO); the common codebook was created incrementally, using triangulation to compare the original (country-specific) codebooks. During the familiarisation process, the researchers found that the vast majority of third and fourth level themes (and the topics they were covering) were so specific to a particular country that cross-country standardisation would not be meaningful (e.g., financing themes *obligatory* and *additional health insurance* in Slovenia; *pay for quality* and *corporatism* in Belgium; and *donor-dependence* in Cambodia). Therefore, these themes were removed, and it was decided to adopt a two-level structure for the common codebook. In subsequent meetings, the content that appeared in all country-specific analyses was extensively examined. Themes that appeared in all three codebooks were first added to a newly formed provisional codebook; then refinement of other themes continued to capture topics that appeared in each country and still allow for unification across codebooks – for instance, themes such as the various organizational structures of primary health care (e.g., family medicine practice, general practice, nursing home, community health care, and home care) were merged into a singular theme called *Primary health care level*. Similarly, the level 2 theme of health care workforce collaboration was relocated from the *Health workforce* theme to the *Collaboration/Communication* theme. This was an iterative process of revising (renaming, removing, and moving) themes in the structure until consensus was reached among researchers. Each decision about the use of a particular code (inclusion, application criteria, potential areas of overlap, and position in the structure) was agreed upon by the entire team. Throughout the whole process, a strong emphasis has been placed on unification of the understanding of the themes under study, their content and inclusion of specific codes within the themes.

#### Phase 4: codebook finalization

In the final phase of the codebook development process, the agreed-upon common codebook was refined by adding labels and definitions. Finally, the structure of the codebook was analysed using the *chunking* approach, in which subsets of text are assigned one or more codes and then used to represent the specific context [31]. The result was the final codebook for qualitative data analysis for the evaluation of integrated care of HTN and T2D, which consisted of a total of nine first level and 39 second level themes. The first level included identified core themes that generally allow for coverage of all issues that arise during the coding process and highlight both facilitators and barriers. The second level themes include the more detailed facilitators and barriers. Although certain first and second level themes may not apply universally across diverse research contexts (e.g., varying countries and/or healthcare systems under study, participants selection, data collection methods, research questions, etc.), it is crucial to ensure that any potential issues arising during the analysis can be appropriately coded using the codebook’s first and second level themes. This approach facilitates the extraction and comparison of results from diverse research endeavours. Table 2 shows the comprehensive codebook with the definition of each theme. The first level theme *Pharmaceutical* does not include second level themes because none of them emerged during the coding process.

**Table 2:** Comprehensive codebook for evaluation of integrated care for arterial hypertension and type-2-diabetes. HTN – arterial hypertension, T2D – type-2-diabetes [33].


THEME	DEFINITION

1. Governance	the process of establishing and implementing principles, structures, and policies to effectively guide and regulate the actions and decisions within a particular system or organization

1.1. Leadership, accountability and management	the skills, responsibilities, and strategies required to inspire, guide, and oversee individuals and resources towards achieving goals while upholding transparency and taking ownership for outcomes.

1.2. Decision-making process	systematic series of steps to making a choice or taking a course of action

1.3. Policy, regulation, strategy	national policy, strategy, and guideline for the prevention and control of HTN and T2D

1.4. Regional and local authorities	designated authority/-ies that has the right to make decisions in an organised political community or in any area of activity at the local level

1.5. Macro-level stakeholders	individuals, groups, or organisations directly or indirectly involved in the macro-level decision-making process (e.g., chambers, national institute of public health, ministry, etc.)

1.6. Stakeholder collaboration	active engagement and cooperative efforts among diverse individuals or groups with a vested interest, fostering shared decision-making and collective problem-solving

1.7. Political interest, commitment and power dynamics	influence, motivations, and actions of political actors within the health care system, highlighting their roles in shaping health care policies, resource allocation, and decision-making processes, which can impact health outcomes and the distribution of healthcare services.

2. Health financing	strategies, mechanisms, and policies employed to generate and allocate financial resources for the provision of healthcare services, ensuring equitable access, sustainability, and efficient utilization of funds

2.1. User financial payment	the amount that is not reimbursed by insurance and is thus paid by the patients (out-of-pocket)

2.2. Budget/Sources of funding	the allocation and management of financial resources to support and sustain operations, projects, or initiatives

2.3. Service provider financial payment	health care system financing (e.g., state budgets, municipal budgets, public health insurance, public pension insurance, voluntary health insurance, non-profit agency budgets, corporate budgets, etc.)

2.4. Health insurance and social protection	establishment of comprehensive coverage and support systems to safeguard individuals and populations against health-related risks, providing financial protection and ensuring access to necessary healthcare services.

3. Organisation of health care	design, structure, and coordination of healthcare services, aiming to optimize the delivery, accessibility, efficiency, and quality of care within a healthcare system

3.1. Primary health care level	foundation level of health care that encompasses essential medical services provided by general practitioners, nurses, and other health care professionals in community-based settings, emphasizing preventive care, health promotion, and basic treatment of common illnesses and injuries

3.2. Secondary and tertiary health care level	level of medical care that involves specialized clinical services provided by medical professionals in hospitals or clinics, focusing on the diagnosis, treatment, and management of specific health conditions and diseases

3.3. Integration throughout the health care continuum	management and delivery of health services to individuals, ensuring they receive a seamless range of health promotion, disease prevention, diagnosis, treatment, disease management, rehabilitation, and palliative care services across various levels and locations within the healthcare system, tailored to their needs throughout their entire lifespan

3.4. Team work	collaborative and coordinated efforts of individuals working together towards a common goal, leveraging their diverse skills, knowledge, and perspectives to achieve optimal outcomes

3.5. Quality of care	ensuring that healthcare services meet the highest standards of safety, effectiveness, efficiency, equity, and patient-centeredness, resulting in improved health outcomes and patient satisfaction

3.6. Following guidelines, protocols	adherence to established standards, evidence-based guidelines, and predefined protocols to ensure consistent, safe, and effective delivery of care and treatment to patients

4. Health workforce	individuals involved in providing healthcare services, including professionals, support staff, and volunteers, who contribute to the promotion, prevention, treatment, and management of health conditions

4.1. Health care workers/Non-health care workers	individuals employed in various roles within health care system

4.2. Administration	management and oversight of organizational, financial, operational, and logistical aspects to ensure the efficient functioning and coordination of healthcare services and resources.

4.3. Time burden	excessive time constraints and pressures experienced by health care providers (e.g., workload, administrative tasks, and limited resource availability)

4.4. Burnout	physical, emotional, and mental exhaustion, as well as the feelings of depersonalization and reduced personal accomplishment, resulting from chronic work-related stress and overwhelming demands within the healthcare profession.

4.5. Task sharing (formal and informal) among team members	formal and informal distribution of responsibilities, duties, and tasks within a healthcare team

4.6. Education, training	acquisition and development of knowledge, skills, and competencies through formal and informal learning processes, aiming to enhance the professional capabilities and performance of health care practitioners

5. Patients	individuals seeking or receiving health care services

5.1. Patients’ attitude	emotions, beliefs, behaviors, and perceptions exhibited by individuals towards their health care experiences, providers, and treatment

5.2. Patient empowerment	process of equipping individuals with the knowledge, skills, and confidence to actively participate in their health care decisions, take control of their health, and collaborate with health care providers for improved outcomes

5.3. Lifestyle	habits, behaviors, choices, and activities individuals engage in daily that can influence their overall health, well-being, and susceptibility to certain diseases or conditions

5.4. Accessibility	availability of health care services, facilities, and information, ensuring that individuals can obtain timely and appropriate care (e.g., proximity of health care facilities, affordability of services, availability of transportation, and language interpretation services)

6. Community actors/Community link	engagement, collaboration, and integration of various individuals, organizations, and resources within the community to support and strengthen health care delivery, health promotion, and addressing local health needs

6.1. Patients’ associations	formation of organized groups or associations by patients or their advocates to collectively advocate for their rights, provide support, share experiences, and promote initiatives aimed at improving the quality of care and the well-being of individuals facing specific health conditions or challenges

6.2. Individuals	patients’ individual connections in the community (e.g., spouses, parents, children, friends, neighbours, etc.)

6.3. Informal caregivers	unpaid individuals who provide physical, emotional, or logistical support to individuals with health conditions or disabilities, playing a vital role in their daily care and overall well-being (e.g., volunteers, patients as teachers, etc.)

6.4. Local community	geographic area and its residents, emphasizing their involvement, resources, social networks, and collective efforts in promoting health, addressing health care needs

6.5. Community health workers	trained individuals who are selected from the local community and work as frontline health care providers, delivering essential health care services, health education, and outreach initiatives to community members, particularly in underserved areas. (e.g., peer educators)

7. Collaboration, communication	coordinated and effective exchange of information, ideas, and resources among healthcare professionals, patients, and stakeholders

7.1. Horizontal and vertical collaboration	coordination among different organizations at the same level of health care or within the same sector (horizontal); and coordination across different levels of care or sectors (vertical)

7.2. Between professionals and laymen	collaboration between health care workers and laymen

7.3. Inter-generational	collaboration between different generations

7.4. Intersectoral	collaboration and cooperation among different sectors, such as healthcare, education, social services, and government

8. Pharmaceutical	development, manufacturing, distribution, and utilization of medications and medical products, with a focus on ensuring their safety, efficacy, accessibility, and appropriate use to improve patient health outcomes

9. Health information system	collection, management, storage, analysis, and dissemination of health-related data and information, supporting decision-making, healthcare delivery, and public health interventions

9.1. E-health	digital technologies, information, and communication systems to support and improve health care delivery, access to medical information, and the overall management of health

9.2. Data management system	organization, storage, processing, and utilization of health-related data and information in a structured and secure manner, ensuring accuracy, accessibility, and confidentiality for effective decision-making and healthcare delivery

9.3. Fragmentation	disjointed and disconnected nature of data and information within healthcare systems, hindering effective data sharing, interoperability, and comprehensive analysis


## Results

We present a codebook which could serve researchers in their first step in deductive or deductive-inductive qualitative research analysis that allows for the organisation of concepts and themes extracted from qualitative data (Table 2) [28,32]. It defines codes and themes by providing detailed descriptions and constraints for inclusion in a code. The codebook is publicly available in repository Open Science Framework [33].

The codebook offers a tool to conduct implementation research on integrated care for HTN and T2D in various health care systems contexts, including high and LMICs. It provides a comprehensive guide for analysing the micro, meso, and macro levels of integrated care. To achieve a more detailed analysis at each level, other frameworks, such as those for patient-centred care or specific health system components, may be used in conjunction with this codebook. Additionally, this tool could complement quantitative frameworks and evaluation instruments for integrated care assessment (e.g., *Assessment of Chronic Illness Care form, Assessment of Innovative Care for Chronic Disease framework tool*, or *Integrated Care Package Grid*) [13,34,35]. It is designed for qualitative analyses and can be utilized for both primary data analysis, including in-depth semi-structured interviews, focus groups, and observations, as well as secondary analysis, such as document analysis. However, it is essential to possess adequate knowledge and skills in qualitative research to use this tool effectively.

## Discussion

This paper presents a codebook for qualitative analysis of integrated care of HTN and T2D that can be used in multiple health care systems across high and LMICs. The experience of developing the common codebook showed us, as predicted, that some themes were universal in all three countries, namely *Governance, Health financing, Organisation of health care, Health workforce, Patients, Community actors/Community link, Collaboration/Communication, Pharmaceutical* and *Health information system*. Therefore, unifying these themes was not difficult, and their relevance is to be expected in other contexts as well. However, other, more country-specific themes were scattered throughout the country-specific codebooks. For example, differences in political structure, health care organisation, financial reimbursement models, and the role of the community in three countries were factors that resulted in a variety of second level themes. The main challenge was to combine and adapt these themes, especially because special care had to be taken to design them in a way that would capture other contexts. Due to the specificity of each context, three and four level codes were ultimately excluded from the final codebook because generalisation wasn’t possible at this stage.

Although the implementation of HTN and T2D integrated care has been qualitatively extensively studied worldwide, and therefore many different codebooks have been developed and used in the analysis processes, the codebook presented in this paper has many advantages. First, unlike many other similar studies that focus mainly on the view on the perspectives of only one or two different stakeholder groups (e.g., patients and health professionals) on the integrated care of HTN and T2D [12,15,36,37,38,39], our study used a broader sample of different stakeholders at the micro, meso, and macro levels. Consequently, their different perspectives on the topic due to their different (professional) backgrounds and entry points into the health care process helped to broaden the scope of the codebook. Second, it allowed the use of different data collection methods. Semi-structured interviews provide in-depth insight into the perceptions of a particular stakeholder. Focus groups, on the other hand, allow for a broader coverage of research topics, as different participants complement each other during data collection, leading to new themes. By triangulating the aforementioned approaches, completeness of the final product could be achieved. Third, this codebook has been co-developed by researchers in different health systems in high and in LMICs. It is thus relevant and applicable in quite varied settings, and facilitates reciprocal learning across contexts, and international collaboration for implementation research.

Our codebook bears partial resemblance with themes in other papers on the topic of integrated care for HTN and T2D. For example, Lemmens et al. identified the themes *Motivation, Competences, Methods* and *Resources* in a qualitative analysis of patient involvement in T2D care [38]. Sims Gould et al. drew on the themes *Education, Social support, Setting (ease of location, ease of conversation)*, and *Impact* when evaluating team-based care for patients with T2D [40]. Ameh discussed the issues of structure-based dimensions (*Accessibility, Supply of medication*, and *Availability of equipment*) and process-based dimensions (*Appointment system, Time with patients*, etc.) of quality of care in the integrated management of HIV and HTN in South Africa [39]. Ide et al. have highlighted numerous barriers and facilitators to diabetes care in Nepal (*General diabetes knowledge, Diet, Physical activity, Medication adherence*, et al.) [9]. The themes that emerged in the aforementioned studies could be easily incorporated into our codebook, making it more suitable for extension and adaptation to new settings.

A limitation of this tool is that it was developed based on HTN and T2D care. Despite variations in integrated care concepts and themes across different health conditions, it is likely that this codebook could be readily adapted for use to other chronic conditions. As a component of our work within the SCUBY project, we were able to successfully modify this codebook to examine macro-level barriers to integrated care, regardless the specific disease.

The presented universality of this tool facilitates reciprocal learning between different health care systems and cross-country lessons; and can therefore support international research on integrated care. The identification of barriers and facilitators supports subsequent recognitions of pathways to scaling-up of integrated care. We recommend the use of this codebook for the study of HTN and T2D, particularly in qualitative research. We encourage other researchers to use it for mixed methods studies as well; moreover, it could be also used as a framework for developing quantitative measures (such as questionnaires). Its usefulness has already been confirmed by the use of the codebook in a published study highlighting the differences between facilitators and barriers to integrated care for non-communicable diseases in Slovenia and Belgium [14]. To our knowledge, there is no comparable instrument on the research topic in the literature to date.

## Conclusion

This article provides a codebook for qualitative assessment of implementation of integrated care for HTN and T2D. We examined the validity and usefulness of the instrument in three very different countries. The codebook is a useful analytical tool for implementation research examining integrated care for HTN and T2D in different settings, facilitating joint learning across settings and countries, advance global implementation of integrated care.

## Additional File

The additional file for this article can be found as follows:

10.5334/ijic.7691.s1 Appendix. Appendix 1 to 3.
