# Supplementary material for: Development of a Qualitative Data Analysis Codebook for Arterial Hypertension and Type-2-Diabetes Integrated Care Evaluation
Source: Int J Integr Care. 2024 Mar 22;24(1):20. doi: 10.5334/ijic.7691 (PMC10959140; doi:10.5334/ijic.7691)
Supplement: Appendix. — Appendix 1 to 3. [file ijic-24-1-7691-s1.pdf]

APPENDIX 1. Thematic guide for semi-structured interviews.

Introduction
Introduction of the researchers:<br><br>1. Name and function of researchers<br>2. Share researchers' contact details (e.g., business card)<br><br>Introduction of SCUBY:<br><br>3. SCUBY is a four-year research project on the scaling-up of integrated care for type-2-diabetes and hypertension through co-creation with key stakeholders, such as you. We are studying the scaling up of integrated care for type-2-diabetes and hypertension, with special attention to vulnerable people.<br>4. The aim of this research is to engage with key stakeholders at all levels, to identify opportunities and barriers to integrated chronic care, implement and support best practices on a larger scale.<br>5. Key terms: integrated chronic care, type-2-diabetes, hypertension, and vulnerable groups<br><br>Explain purpose and the intent of the interview:<br><br>● Aim of interview: gaining insight into your position in relation to integrated care and current initiatives<br>● Rationale for selection<br>● Duration of interview (up to 90 minutes)<br>● Ensure anonymity and confidentiality<br>● Questions?<br><br>Informed consent:<br><br>● Ask (to sign) the informed consent and permission to record the interview<br><br>Outline of interview (topics to cover):<br><br>6. Integrated chronic care (ICC)<br>7. Policy & practice<br>8. Stakeholders<br>9. The financing system

10. Remarks and conclusion
I. Warm-up questions
On the position of the representative within his/her organization:<br><br>Q#1.1 - Can you briefly describe your position and role within your organization?<br><br>On the organization:<br><br>Q#1.2 - Can you shortly describe the main goals and main activities of your organization?
II. Integrated chronic care (ICC)
General understanding of integrated chronic care:<br><br>Q#2.1 - What is your **understanding** of integrated chronic care? (What are key elements in your experience?)<br><br>Q#2.2 - How is integrated care relevant to type-2-diabetes and hypertension care?<br><br>Q#2.3 - Which of these categories best describes **your opinion** on integrated care policy for type-2-diabetes and hypertension? (Read the options and circle the answer given.)<br><br>a) I strongly support it<br><br>b) I somewhat support it<br><br>c) I do not support nor oppose it<br><br>d) I somewhat oppose it<br><br>e) I strongly oppose it<br><br>Q#2.4 - What kind of **potential benefits do you see for your organisation** of integrated care for type-2-diabetes and hypertension patients?

Q#2.5 - What do you think that are potential disadvantages/difficulties/threats for your organisation of integrated?

Q#2.6 - How do you see **your role** in the current state of implementation of the integrated care?

Q#2.7 - How do you think that the current system can move towards integrated chronic care in your country? Which **strategies** can be used? Would you or your organisation be interested in taking the initiative in this move?

Define Integrated Care Package

Notes:

III. Policy and practice

Policies:

Q#3.1 - Which/where are according to you the **barriers and challenges** for better care integration?

- In the political system?
- In the decision making processes?
- According to the division of responsibilities at different levels?
- In the health care financing system?
- Information technology?
- Available resources?
- Available competences?
  - Local managers/governance?
  - Care providers?
  - Patients?
- Other aspects of health care system?

Resources:

Q#3.2 - What are the resources you have available (human, financial, technological, political, etc.)?

Q#3.3 - How quickly can these resources be mobilised? What is the main hurdle and what can you do

to optimize care?<br><br><br>Best practices:<br><br>Q#3.4 - Are there any **examples of policies/projects** that were (in your view) successful in improving integrated care for type-2-diabetes and hypertension patients? What are the **'best practices'**? **Why** were they successful? **What can we learn** from these examples? Are those projects still alive or why do you think that they didn't manage to stay part of the system?
IV. Stakeholders
Stakeholders:<br><br>We would now like to ask you a few specific questions about your opinion regarding others' opinions of the implementation of the integrated care policy.<br><br>Q#4.1 - Who are **the key stakeholders** in your view? Whose role is **central** in the achievement of ICC [within a certain policy]? Who should be **in charge** of which reforms?<br><br>Q#4.2 - How would you describe your **collaboration** with or relation to the key stakeholders? How they interact with other caregivers and social organisations? Who do you work closely with?<br><br>Q#4.3 - What other organizations, departments within an organization, or persons do you think would **support** ICC-policy?<br><br>Q#4.4 - What do you think these supporters would **gain** from ICC-policy?<br><br>Q#4.5 - Which of these supporters would **take the initiative** to actively support ICC-policy?<br><br>Q#4.6 - What other organizations, departments within an organization, or persons do you think would **oppose see difficulties in** ICC-policy?<br><br>Q#4.7 - What do you think these opponents would gain from preventing ICC-policy? Why would they be opposed?<br><br><br>Vulnerable people:

Q#4.8. - What are vulnerable populations in your view? How are they identified?<br><br>Briefly explain how we define vulnerable people in SCUBY.<br><br>Q#4.9 - How do you reach vulnerable people?<br><br>Q#4.10 - What do you see/think that is the biggest obstacle for vulnerable groups to get the proper care?
V. The financing system
Q#5.1 What do you think about the current health financing arrangements – what are strengths and weaknesses? Do you have any recommendations to address the weaknesses?
Concluding remarks
Conclusion:<br><br>Q#6.1 On a scale from 1 (meaning no integrated care) to 10 (meaning fully integrated care), how far do you think we are in this country in the transition to integrated chronic care? Can you also briefly say why you have given that score?<br><br>Additional comments:<br><br>● Do you have any additional remarks?<br>● Is there something that you think we didn't cover that is still relevant to this issue/topic?<br>● Is there someone else you think we should talk to, that you can identify as a key stakeholder?<br><br>We'll be analysing the information you and others gave us and submitting a draft report in a few months. I'll be happy to send you a copy to review (our findings) at that time, if you are interested.<br><br>Share SCUBY brochure at the end.

APPENDIX 2. Thematic guide for focus groups.

Introduction
Introduction of the researchers:<br><br>• Name and function of researchers<br><br>Introduction of SCUBY:<br><br>• SCUBY is a four-year research project on the scaling up of integrated care for type-2-diabetes and hypertension. We are studying the scaling-up of integrated care for type-2-diabetes and hypertension, with special attention to vulnerable people.<br>• The aim of this research is to engage with stakeholders at all levels, including patients and healthcare staff, to identify opportunities and barriers to integrated chronic care and to support and implement best practices on a larger scale.<br>• Key terms: integrated chronic care, diabetes, hypertension and vulnerable groups<br><br>Explain purpose and the intent of the interview:<br><br>• Aim of interview: gaining insight into opinions and perceptions of patients, health care teams and community actors in relation to integrated care, its barriers and facilitators<br>• Duration of focus group interview (up to 90 minutes)<br>• Ensure confidentiality<br>• Questions?<br><br>Informed consent:<br><br>• Ask (to sign) the informed consent and permission to record the interview
A. Questions to doctors/health care staff/teams

Opinion on current process:

- Which adaptations were made at the implementation of the integrated care package at the health care facility and at other organisations involved?
- Which external organisations were involved to support implementation?
- How were health workers, other actors and patients prepared for implementation?
- What was the role of local and central health authorities in the implementation?

Facilitators:

- In your opinion, what is good in the current implementation of the integrated care for people with type-2-diabetes and hypertension?
- How are vulnerable population identified? How well does ICP reach vulnerable people?
- What are important factors that facilitate the current way of working? What are further options for improving management (detection, treatment, and self management) of people with type-2-diabetes and/or hypertension?Especially regarding vulnerable people?
- How do you see the directions of possible extension of care/scaling-up and facilitators to that?
- Who are potential people/approaches that can be added to the existing care?
- How do you see the role of patients as teachers? How do you see the role of organisations in the community? How do you see the role of informal caregivers?

Barriers/obstacles:

- What are your personal experiences/what kind of obstacles (in screening, testing, treatment) have you considered in the ICP-process (screening, testing, diagnosing, retaining in care, follow-up, self management, different levels cooperation) for (vulnerable) patients with type-2-diabetes and/or hypertension at the primary level?
- What are the problems of people who don't come regularly for care? What are current actions you take? What are other options?
- Where are the obstacles that would prevent expansion or change in the process?

Care and financial barriers:

- What according to you are major financial barriers to appropriate health care for patients with type-2-diabetes and/or hypertension treatment?

B. Questions to patients

Opinion on current process:

- Where are you seeking/gaining the most knowledge and support/what kind of self-management does it help you/do you use?
- Where do you (or your family members) usually go for care for type-2-diabetes/hypertension?
- Are there other people you go to for information about type-2-diabetes/hypertension apart from your doctor?
- Are there people in the environment who help you in the management of your type-2-diabetes/hypertension?
- Are there organisations outside of the hospital that are of help in the management of your disease?

Facilitators:

- What are your personal experiences with the diagnosis/treatment and follow up of your disease?
- What is good in the existing process (about the treatment of your disease, follow-up, communication, and shared decision making with health care workers, plan care tailored to your needs and circumstances)?
- of patients as teachers? How do you see the role of organisations in the community? How do you see the role of informal caregivers? Who are potential people/approaches that can be added to the existing care?
- In what way do you see the care to develop in order to give you the greatest support and to gain the most out of it?

Barriers:

- What do you feel is not so good in the process? What can be changed?
- Where are the obstacles that would prevent expansion or change in the process?

Care and financial barriers:

- Has your lifestyle changed since being diagnosed? How?
- Describe your treatment. On which items you spend the most money for your treatment? Have there been any changes in your health expenditure since you were first diagnosed? What about expenditures on transportation, special food, special health activities?
- What kind of home equipment for self-care does your condition require?
- Are there some services available which could be useful but unaffordable or difficult to access? Do you know people who were able to get access to such services? How do they manage? Do you/did you face barriers to access the services/getting care? Do you know other people who should come to the services, but don't come? What are the reasons for this?
- Are there any other financial views (not really barriers) which could help you to better manage your condition?
- Can you please describe what you do on your own for your health and better management of your disease?

C. Questions to community actors (Cambodia)

Facilitators

- In your opinion, what is good in the current implementation of the integrated care for people with type-2-diabetes and hypertension?
- What is your work/communication flow with the people living in the community and Health Center Management Committee (HCMC)?
- How well do you reach and engage with people in the community?
- What are important factors that facilitate the current way of working?
- What are further options for improving community engagement in the integrated care of people with type-2-diabetes and/or hypertension?
- How do you see the directions of possible extension of care/scaling up and facilitators in doing that?

• Who are potential people/approaches that can be added to the existing care?<br><br>Barriers:<br><br>• What are your personal experiences/what kind of obstacles in your work as the community health worker? Working flow with community and HCMC?<br>• What are the problems of people who don't come regularly for care? Don't take action with your message/advise? What are current actions you take? What are other options?<br>• Where are the obstacles that would prevent expansion or change in the process?<br><br>Care and financial barriers:<br><br>• Do you provide specific services to the community (especially peer educator such as offer blood glucose/blood pressure measurement)? How is the service charged? How is the affordability and accessibility of this services to patients in the community?<br>• What according to you are major financial barriers to appropriate health care for patients with type-2-diabetes and/or hypertension treatment from your experience engaging with patients?
Concluding remarks
Additional comments:<br><br>• Do you have any additional remarks?<br><br>Share SCUBY brochure at the end.

APPENDIX 3. Detailed selection of participants.

Participants level	Cambodia	Slovenia	Belgium
Micro (focus groups)	Patients – Siem Reap, Takeo, and Prey Veng (4 focus groups)<br><br>Community Health Workers – Siem Reap, Takeo, Kompong Speu, Oddormeanchey, and Prey Veng (5 focus groups)<br><br>Healthcare staff – Siem Reap, Takeo, Kompong Speu, Oddormeanchey, and Prey Veng (5 focus groups)	Patients - Ljubljana (6 focus groups)<br><br>Patients - Ravne na Koroškem<br><br>Healthcare workers - Ljubljana (5 focus groups)<br><br>Healthcare workers - Lendava<br><br>Healthcare workers - Gornja Radgona<br><br>Healthcare workers - Ravne na Koroškem	not performed
Meso (semi-structured interviews)	Representative of the Operational District (5 interviews)<br><br>Representative of the Provincial Health Department (5 interviews)<br><br>Representative of the Referral Hospital (5 interviews)	Community Health Centre Ljubljana (3 interviews)<br><br>Community Health Centre Postojna<br><br>Institution for informal home care<br><br>(Zavod za oskrbo na domu)<br><br>Municipality of Ljubljana (2 interviews)	Medical Association of GPs (Domus Medica, DM)<br><br>Belgian Association of Doctors Syndicates (BVAS)<br><br>Medical Association of GPs and Specialists (ASGB)<br><br>General Pharmaceutical Association (APB)

		Associations of patients with chronic diseases<br><br>(Društvo Za Srce) (2 interviews)<br><br>Retirement home (Dom starejših občanov Tabor)<br><br>Associations of patients with chronic<br><br>diseases (Društvo diabetikov)	Network of Hospitals (ICURO)<br><br>Flemish Association of Dieticians<br><br>Association of Diabetes Nurses<br><br>Flemish Association of<br><br>Independent Nurses (VBZV)<br><br>Network of Homecare Nurses (Zorggezind)<br><br>Association of Home Nursing (WGK)<br><br>Flemish Patient Platform (VPP)<br><br>Flemish Diabetes Association (Diabetes liga)<br><br>First line zone (3 zones: Antwerp,<br><br>Ghent, Kempenland)
Macro (semi-structured interviews)	Department of Preventive Medicine, Ministry of Health (3 interviews)<br><br>Department of Hospital	National Institute for Public Health (2 interviews)<br><br>National Institute for Health Insurance	Federal Public Service of Health (FOD)<br><br>Flemish Cabinet<br><br>Association of Flemish

	Services, Ministry of Health<br><br>Department of Planning and Health Information, Ministry of Health<br><br>Department of Human Resources<br><br>National Center for Health Promotion<br><br>Payment Certification Agency<br><br>Nacional Social Security Fund<br><br>MoPoTsyo Peer Education Network<br><br>World Health Organization, Cambodia office<br><br>Louvain Cooperation, Cambodia office<br><br>World bank, Cambodia office<br><br>GIZ (Deutsche Gesselschaft für Internationale Zusammenarbeit), Cambodia office	Ministry of Health (2 interviews)<br><br>Chamber of Pharmacies<br><br>Health Council at Ministry of Health<br><br>Chamber of nurses, midwives and<br><br>healthcare assistants of Slovenia<br><br>Medical University of Ljubljana, Department<br><br>of Family Medicine (3 interviews)<br><br>Medical Chamber	Cities<br><br>and Municipalities (VVSG)<br><br>Flemish Agency of Care and Health (VAZG)<br><br>National Institute of Health & Disability<br><br>Insurance (NIHDI) (3 interviews)<br><br>Christian Health Fund (CM)<br><br>Joint College of Sickness Funds (NIC)<br><br>Socialist Sickness Fund<br><br>Federal Knowledge Centre for Healthcare (KCE)<br><br>Academia/Medical universities (2 interviews)

	University Research Co., LLC, Health and Social Development Project<br><br>University of Health Sciences<br><br>Sihanouk Hospital, Center of Hope<br><br>Preah Kossamak Hospital		
